# Perinatal Loss and Parents’ Grief Amidst the COVID-19 Pandemic: A Mixed-Method Research

**DOI:** 10.3390/bs14040339

**Published:** 2024-04-18

**Authors:** Ciro De Vincenzo, Loredana Cena, Alice Trainini, Chiara Nieddu, Erika Iacona, Lucia Ronconi, Ines Testoni

**Affiliations:** 1 Department of Philosophy, Sociology, Education and Applied Psychology, University of Padua, 35122 Padua, Italy; ciro.devincenzo@unipd.it (C.D.V.); niedduchiara2@gmail.com (C.N.); erika.iacona@studenti.unipd.it (E.I.); 2 Department of Clinical and Experimental Sciences, Section of Neuroscience, University of Brescia, 25123 Brescia, Italy; loredana.cena@unibs.it (L.C.); alice.trainini@unibs.it (A.T.); 3 Computer and Statistical Services, Multifunctional Pole of Psychology, University of Padua, 35122 Padua, Italy; l.ronconi@unipd.it; 4 Emili Sagol Creative Arts Therapies Research Center, University of Haifa, Haifa 3498838, Israel

**Keywords:** perinatal death, perinatal loss, grief and bereavement, parents, COVID-19

## Abstract

Losing a child is a traumatic event, disrupting life’s natural cycle, profoundly affecting the family system, and causing enduring grief. Perinatal death, including ectopic pregnancies, miscarriages, stillbirths, and neonatal deaths, exacerbates this distress. Additionally, the COVID-19 pandemic has challenged healthcare systems and supporting services available to individuals in need. Thus, this research explores experiences of parents facing perinatal loss in 2020–2021, further focusing on the pandemic’s impact. Using a mixed-methods design with self-reports and qualitative interviews, this paper presents results from the quantitative protocol, involving an update and follow-up of a previous study. It compares measurements across scales: COVID-19: The Impact of Event Scale-Revised; The Prolonged Grief-13; The Parental Assessment of Paternal/Maternal Affectivity; The Dyadic Adjustment Scale (short version); The Daily Spiritual Experiences Scale; and The Inventory of Complicated Spiritual Grief. In the baseline measurement, 45 parents participated (37 mothers and 8 fathers), with 20 (13 mothers and 7 fathers) contributing to the follow-up and 9 engaging in interviews. Baseline results showed higher scores for mothers compared to fathers, with effect sizes ranging from small to medium (ranging from −0.02 to 0.29), though statistical significance was limited due to the small sample size. Multiple regression analysis for distress measures at baseline identified two significant predictors: maternal/paternal affectivity and gestational week. Additionally, positive support from healthcare professionals emerged as a mitigating factor, particularly in relation to Avoidance. A significant reduction in stress measures and parental affectivity was observed at the 6-month follow-up. Qualitative analysis revealed three themes: Shifts in Self-Perception and Post-Loss Growth; Conflicted Relationship with One’s Body; and Negative Impact of COVID-19 vs. Unexpectedly Positive Aspects. In conclusion, the findings emphasize the significance of psychological and psychosocial interventions based on meaning-making processes, along with the importance of spiritual care and empowerment for those navigating perinatal loss.

## 1. Introduction

The loss of a child is an intensely dramatic event that disrupts the natural course of life [1,2,3,4,5]. It deeply affects mothers, fathers, and the entire family system, potentially leading to intense and enduring grief that persists for several years [6]. While grief is a normal response to the death of a loved one, it can lead to complicated grief [7] or prolonged grief disorder [8], particularly when the loss occurs in sudden or violent circumstances [9]. Particularly, perinatal death encompasses various form of loss such as ectopic pregnancies, miscarriages, stillbirths, and neonatal deaths [10]. A significant number of women experience complicated grief and post-traumatic stress following perinatal death [11,12]. The sudden and unexpected nature of the death represents an additional burden and challenge for families facing perinatal death [13]. Furthermore, perinatal bereavement is a multifaced experience that involves multiple stressors, including a threat to the personal meaning of motherhood [14], the profound disruption to parental aspiration [15], and the absence of a transgenerational imagined future [1,2]. However, despite its traumatic potential, perinatal loss is often invisible and silenced [12]. Indeed, perinatal grief is among the losses characterized by social delegitimization and thus identified as a form of disenfranchised grief [16]. Literature indicates that women experiencing Intrauterine Fetal Demise (IUFD) are at higher risk for the diagnostic conditions post-traumatic stress disorder (PTSD) and depression, compared to women whose babies are born alive [17]. Moreover, IUFD carries an even more stressful dimension due to its “embodied” nature, occurring within the maternal body [3,4]. In addition, women may also grapple with the failure of their bodies and perceive a rupture of their femininity [13]. In some cases, they may report feelings of shame and guilt about their failure as caregivers [18]. Additionally, the loss of a child during pregnancy can strain the relationship with the partner [19]. Unequal or incongruent grieving between partners is considered as a major risk to relationship well-being and satisfaction [13]. Globally, according to the Lancet, stillbirth accounts for approximately 2–6 million deaths per year in 2016 [20,21,22]. These data underscore the impact of the phenomenon, defining perinatal death as a major public health concern [23]. Specifically, the World Health Organization reported that in 2019, Italy had a stillbirth rate of 2.39 per 1000 births.

In the context of the COVID-19 pandemic, maternity care has undergone significant changes and disruptions, affecting various aspects of women’s lives during pregnancy, delivery, and the postnatal period [24,25]. The literature confirms that levels of stress, anxiety, and depression among pregnant women have increased during the pandemic [26]. For instance, partner’s absence during and after childbirth due to COVID-19-related hospital restrictions has proved to be a relevant factor in increasing mothers’ psychological distress [27]. A recent study revealed that mothers separated from their partners exhibited higher levels of anxiety compared to those who were not and lower perceived caregiver support [28]. Moreover, a study conducted by Durankuş and Aksu in 2020 [29] involving 260 pregnant women who completed anxiety and depression assessment scales online found that symptoms were twice as high as those observed under normal circumstances. This highlights the impact of the transitional nature of the perinatal period, which can lead some women to experience a heightened distress and vulnerability. Additionally, in a recent study by Shuman and colleagues (2022) [30], some of the participants also reported feelings of guilt due to the restrictions caused by the COVID-19 pandemic, and of inadequacy and responsibility for the altered newborn experiences. The increased risk of anxiety and depression during the perinatal period exposes new mothers and pregnant women to an even higher risk of psychological stress during the pandemic period.

Moreover, the spiritual dimension and religiosity can facilitate a deeper understanding of individuals as a whole. Indeed, these dimensions can foster connections with oneself, others, nature, and God or the divine [31]. Typically, people rely on religion and spirituality to cope with periods of high emotional stress and declining health [32]. Moreover, the spiritual/religious dimension aids in finding meaning amid the most difficult and challenging circumstances [33], such as the traumatic experience of the COVID-19 pandemic [33,34]. In particular, those grieving may find comfort in viewing death as a passage and in maintaining a bond with the departed [35], often managing anger more effectively than those who feel abandoned by a higher power [33]. What Testoni and colleagues [35] highlighted in their study is that cancer patients often use a more abstract language associated with the concept of God, which results in lower levels of anxiety and distress. Since abstract terms refer to the psychological dimension, the use of an abstract vocabulary can be read as a way for individuals to translate the feeling of greater psychological closeness to God. However, Burke and Neimeyer (2013) [36] found that the death of a loved one can also trigger a spiritual struggle. Neimeyer and colleagues (2021) [37] found that survivors of violent and traumatic loss may exhibit negative religious coping and depression. Higher levels of depression were associated with increased spiritual struggle, particularly in cases where the relationship with God was perceived as lost or damaged. This underscores the significance of spiritual struggle as a potential indicator of the challenges of post-loss “recovery”. Burke and Neimeyer (2013) [36] explored, through a qualitative focus group analysis, the possible spiritual crisis of bereaved people, defining spiritual crisis as an experience consisting of an inability to give a spiritual meaning to the loss. In other words, there is a significant change in spiritual beliefs and behaviors. It is often accompanied by the presence of negative feelings against God and concern about the afterlife and its representation. To evaluate this phenomenon, they developed the “Inventory of Spiritual Complicated Grief”, which measures spiritual crisis in mourners.

Since the number of studies on the topic is lacking, the following research presents a mixed-method on the psychological impact of perinatal loss, which is the loss of a child before their birth, during the COVID-19 pandemic. Indeed, it is significant to promote a comprehensive and multi-level understanding on the psychological expected outcomes of perinatal death as well as on how parents deal with the stressful event. Moreover, addressing the developmental nature and changes over time of such a loss could enhance more suited clinical interventions, tailored to specific challenges.

Specifically, the research aimed to investigate the experiences related to the impact of the COVID-19 pandemic on mothers’ and fathers’ grief who experienced perinatal loss during 2020 and 2021. In particular, the aim was to investigate the impact of COVID-19 on the experience of the parental couple six months after the baseline phase of the study by comparing the narratives of mothers and fathers (for preliminary results on the baseline phase cfr. [5]). Changes over time in the grieving process were assessed at the baseline and the follow-up in order to check for the evolution or development of the grief reaction and experience. Correlations of grief with anxiety, depression, couple satisfaction, and spirituality were detected. Finally, the study investigated which psychosocial factors negatively influence the grieving process and which factors are predictive of the development of complicated grief. For example, it was analyzed how the convergence of traumatic experiences can lead to a crisis in faith, thus reducing the chances of turning to religion as a protective factor. The main hypothesis is that in mothers, the loss may generate greater trauma, signaled by the presence of symptoms of anxiety and depression. In addition, it was claimed that more negative outcomes in processing are related to a difficult couple relationship.

## 2. Materials and Methods

### 2.1. Study Design

A mixed-methods design was adopted through the use of a self-report protocol [38] and the implementation of a semi-structured interview [39]. The longitudinal-type research was divided into a base-line phase [5,38] and a follow-up phase six months after the first one (as the Criterion A for Prolonged Grief Disorders requires a 12-month temporal line, a follow-up after six months was considered a pertinent temporal evaluation). The mixed-method design involves the parallel analysis of quantitative and qualitative data. The choice to adopt a mixed-method research design lies in the aim of triangulating different data (quantitative measurements and subjects’ accounts) and, thus, strengthening the perspective on the subject. Specifically, a mixed-method design was considered particularly relevant to this case as participants did have the possibility to provide more person-centered experiences and feelings regarding their loss. Triangulating and integrating different frames thus enriches the complex view. In the qualitative part, the theoretical framework lies in the narrative paradigm, whose sensibility for individuals’ stories and meaning-making process was considered pertinent to research aims. The mixed-method design we report has followed best practices of conducting and presenting results from such design.

### 2.2. Participants and Ethics

In the baseline measurement, 45 parents participated (37 mothers and 8 fathers), with 20 (13 mothers and 7 fathers) contributing to the follow-up and 9 participating in follow-up narrative interviews, all living in northern Italy. Four participants did not attend the interview despite their initial consent. Almost all the mothers defined themselves as Catholic. A similar situation occurred among fathers. Recruitment took place on voluntary choice in health centers throughout Italy by professionals working in health care in collaboration with the Perinatal Clinical Psychology Observatory of the University of Brescia. Participants experienced perinatal loss (meaning that they lost a child prior to their birth due to unexpected medical emergencies) during the pandemic period. The inclusion criteria were the following: the loss occurred from March 2020 to March 2021; being able to speak the Italian language. The exclusion criteria were the following: not undergoing a psychopharmacological treatment for a previous mental health condition (as the focus of the research and, thus, of the protocol could not take into account other relevant mental health conditions). However, as indicated in the previous baseline [38], considering a power (1 − β) of 0.80 and a type I error (α) of 0.05, a sample of 34 parental couples was needed and thus we met these expectations and align with existing studies.

An Information Note with a description of the aims of the study was given to the recruited subject. To participate, the subject was asked to sign an Informed Consent Form. The protection of their privacy was ensured by the assignment of a code and a pseudonym to the participants (for qualitative results presentation purposes). The research followed the APA Ethical Principles of Psychologists and Code of Conduct. All the objectives of the research and the methodology of analysis used were explained to the participants in detail. The study was approved by the Ethics Committee for Experimentation of the University of Padua (n. 53FB052AA456203CE7F4E9C76EBAFAEE).

### 2.3. Instruments

Six months after administration of the self-report measures and adherence to the first interview, participants were invited to a follow-up. As the temporal criteria for prolonged grief disorder has been set at 12 months, a 6-month follow-up is a relevant time to monitor the development of variables. The following instruments were used:

1. A sociodemographic form for the mother/father that captures sociodemographic data (age, marital status, nationality, education, profession, economic status. The economic status was addressed as following: very low: having debts or not being able to pay for rent; low: having trouble in daily expenses; medium: minor difficulties; high: having home ownership; see Table 1), partner information, medical history data (date of delivery, where it occurred, number of pregnancies, any previous abortions) any disorders or problems, social support (family, friends, health services).

2. COVID-19: The Impact of Event Scale-Revised [40], which assesses subjective distress caused by traumatic events by self-assessment of 22 items. Firstly, respondents are asked to identify a specific stressful life event and then indicate the degree of distress they experienced in the following week for each “difficulty” listed [41]. The IES-R is a revised version of the IES and was developed because the original version did not include hyperexcitation as a subscale. The revised version showed as good psychometric properties as the original one. Test–retest reliability (r = 0.89–0.94) and internal consistency (Cronbach’s α) for each subscale (intrusion = 0.87–0.94, avoidance = 0.84–0.97, hyperexcitation = 0.79–0.91) are acceptable. The Cronbach’s alpha of the IES-R in this study is 0.93 for female participants and 0.87 for male participants.
3. The Prolonged Grief-13 (PG-13) [42,43], which assesses the presence of prolonged grief symptoms, analyzes five criteria: the event of loss, separation anxiety, the duration criterion, cognitive, emotional, and behavioral symptoms, and the significant functional impairment in daily life after six months. A prolonged grief diagnosis is given when all five criteria are met. The instrument consists of 13 items, of which 2 are dichotomous and 11 are on a five-point Likert scale. Internal consistency analysis confirms the single-factor structure, with a Cronbach’s α coefficient of 0.93. Examples of items are “How often have you tried to avoid remembering that the person you lost is missing?”, “Have you had trouble accepting the loss?” [7]. The Cronbach’s alpha of the PG-13 in this study is 0.83 for female participants and 0.72 for male participants.
4. The Parental Assessment of Paternal/Maternal Affectivity (PAPA; PAMA) [44], which assess paternal and maternal affectivity, respectively, during the perinatal period using a ten-point Likert scale. Specifically, these instruments are used to investigate the presence of anxiety, depression, perceived stress, anger, relationship problems, behavioral alterations, and physiological and addiction disorders. Examples of items: “I have had difficulty in relating to others or others have had difficulty in relating to me (my partner, family members, friends, at work, etc.)”; “I have had problems sleeping, eating or in my sex life (even just one of these)”. The Cronbach’s alpha of the PAMA/PAPA in this study is 0.68 for female participants and 0.77 for male participants.
5. The Dyadic Adjustment Scale short version (DAS-4) [45], which assesses parental couple satisfaction. It consists of four items, three of which are on a 6-point Likert scale, while the last item is on a 7-point scale. Sample items: “Do you confide in your partner?”; “How often do you and your partner argue”. The Cronbach’s alpha of the DAS-4 in this study is 0.61 for female participants and 0.74 for male participants.
6. The Daily Spiritual Experiences Scale (DSES) [46] is an instrument consisting of 16 items on a 6-point Likert scale that assesses one’s relationship with one’s spiritual and transcendent dimension and the perceived presence of that dimension in one’s life. As confirmed by the literature, the instrument has good reliability (internal consistency estimate 0.90). Sample items: “I feel guided by God in the midst of daily activities”; “I find strength in my religion or spirituality”. The Cronbach’s alpha of the DSES in this study is 0.93 for female participants and 0.96 for male participants.
7. Finally, the follow-up involved the administration of The Inventory of Complicated Spiritual Grief (ICSG) [36], which measures how much the level of loss corresponds to a spiritual crisis, which may relate to one’s relationship with God or members of the religious community. The instrument consists of 18 items with a 5-point Likert response scale (0 = not at all true; 5 = absolutely true). The authors identified a two-factor structure of (a) “Insecurity toward God” and (b) “Disruption in religious practice.” The Cronbach’s alpha of the original total scale is 0.96 [37]. Sample items are: “I no longer feel safe and secure from God”; “I don’t feel much desire to join the community to praise God or glorify him” [47]. The Cronbach’s alpha of the ICSG in this study is 0.76 for female participants and 0.65 for male participants. The self-report questionnaires were uploaded to an online platform. Data were analyzed using IBM SPSS Statistics, Version 29. (see Table 1 for the Cronbach’s alpha of each instrument).

See Table 2 for Cronbach’s alpha at the baseline and at the follow-up.

For the qualitative component of the research, we conducted semi-structured interviews following a narrative methodological approach. Each interview lasted from forty minutes to one hour. The semi-structured interview aimed to explore how participants experienced their personal and social worlds, and how they made sense of their most meaningful experiences. Therefore, qualitative sensitivity can be an important hermeneutic tool through which to understand the complexity of social reality as experienced by participants. The researcher, sensitive to the semantics of life, recognizes the active role of participants in the construction of their reality [48]. Through interviews, researchers seek to understand the complex social aspects related to perinatal bereavement experiences during the COVID-19 pandemic period. In particular, a series of questions was presented on the following topics: the impact of loss on mood, the dynamics of grieving, the relationship with the body and the possibility of having a new child, the impact of anti-COVID-19 vaccinations, the relationship with spirituality and religious activities (examples of questions: How would you describe your relationship with religious and spiritual activities in recent months? What is your relationship with God after the loss of your child?).

### 2.4. Quantiative Analysis

The comparison between mothers and fathers on the total scores of the measures of interest was conducted with the non-parametric Mann–Whitney test due to the small number of fathers’ responses and biserial rank correlation was used as effect size measure. Instead, for the comparison between mothers and fathers on the presence/absence of PTSD, DLP, and distressed spouse, the Chi-square test and tetrachoric correlation as effect size measure were used. We consider 0.10, 0.30, and 0.50 the cut-off values for small, medium, and large effect-size. To evaluate the impact of participants’ characteristics, perinatal paternal/paternal affectivity, couple satisfaction, and spirituality on distress measures at the baseline, multiple regression analysis on each distress measure was conducted. Finally, to compare the follow-up and baseline assessment, we used the paired *t*-test and Cohen’s d as measure of effect size. We consider 0.20, 0.50, and 0.80 the cut-off values for small, medium, and large effect-size.

### 2.5. Qualitative Analysis

The interviews were transcribed verbatim, then analyzed and later translated into English for the purposes of sharing results among the scientific community. More specifically, a reflexive thematic analysis approach (RTA) was used [39] through which two members of the research team (C.D.V. and C.N., who also realized the interviews) independently familiarized and coded data. Indeed, RTA places a strong emphasis to the iterative or recursive analytic strategy, through which the analyst combines a top-down approach (theory-driven) and a bottom-up strategy (data-driven) into an abductive process of analysis where previous knowledge and unexpected discoveries can be dialogically merged. After data familiarization and coding, which represent two of the most important stages of the analytic process since the analysts can gain an overall view of the data, created codes were compared among analysts. Codes belong to the descriptive dimension of the RTA, and they were identified by paying close attention to participants’ words. Subsequently, the two analysts had meetings with the scientific coordinator (I.T.) in order to merge codes into categories/families according to their sensitivity, positionality, and knowledge of the phenomena. Finally, as major dissimilarities in categories were discussed with the whole team, the generation of themes was realized according to research aims as well as previous results from the literature. Thus, themes belong to the interpretative dimension of the RTA, which is built from a closer coding and a subsequent merging of codes into intermediate categories.

## 3. Results

### 3.1. Quantitative Results

The number of participants, mothers and fathers, who filled out the questionnaire in the follow-up was 20 out of 45 of total participants at the baseline ([5], see also Table 2). For 61.5% (50% of the mothers and 20% of the fathers) this was their first pregnancy, and 46% (37.5% of the mother and 60% of the fathers) had previous experience with miscarriage. Only 23% of the group perceived significant support from the health personnel they met. Half of the mothers reported the physical absence of their partner or the unavailability to access medical services due to COVID-19 restrictions. The same result was reported by 40% of the fathers. Finally, all the participants considered support from friends and family members as sufficient.

As Table 3 reports (see Table 3), higher scores were observed for mothers compared to fathers, with effect sizes ranging from small to medium (ranging from −0.02 to 0.29). However, these effects did not reach statistical significance due to the small sample size.

Furthermore, multiple regression analysis for distress measurers at the baseline was performed (see Table 4). Emerging as the most relevant predictors were two factors: maternal/paternal affectivity (higher affectivity is associated with greater distress) and gestational week (higher gestational week is linked to increased distress). A positive role of support received from healthcare professionals is highlighted, particularly concerning Avoidance (greater support associated with lower Avoidance). Additionally, a positive impact of couple satisfaction on Intrusiveness is emphasized (higher satisfaction linked to lower Intrusiveness). A significant association is noted between economic condition and Intrusiveness (medium-high economic condition associated with greater Intrusiveness). Furthermore, an association between occupation and Intrusiveness is observed, indicating that workers experience higher levels of Intrusiveness.

As table five shows (see Table 5), significant reductions in stress, with a strong effect size, were observed, indicating a substantial decrease in overall stress levels. A notable decrease was also noted in maternal/paternal affectivity, with a strong effect, suggesting a considerable decline in emotional response. However, couple satisfaction and spirituality did not exhibit significant changes, remaining relatively stable throughout the analysis period. While there was a decrease in the proportion of “Yes” responses for PTSD and DLP, the changes did not reach statistical significance. This implies a trend towards improvement, although not statistically confirmed. Interestingly, the proportion of distressed spouses remained unchanged, highlighting a consistent level of distress among this subgroup. Overall, these quantitative findings suggest notable positive changes in stress and affectivity, with certain aspects, such as distressed spouses, showing a more stable pattern.

Finally, with the regard to ICGS evaluation in the follow-up, 20% of the participants reported experiencing no spiritual crisis after the loss. Moreover, 65.0% expressed doubts about their spirituality following the loss, and 0.0% described a normal spiritual conflict after the loss. Among them, 5.0% reported undergoing a strong spiritual crisis after the loss. These findings indicate a diverse range of spiritual experiences among the participants, reflecting varying degrees of impact on their spirituality in the aftermath of a loss.

### 3.2. Qualitative Results

#### 3.2.1. Shifts in Self-Perception and Post-Loss Growth

Bereaved parents acknowledge the change in self-perception. In her narrative, Gaia, 39 years old, emphasizes a significant shift in her identity. Two years after experiencing a miscarriage, she describes herself as more serious yet aware of her vulnerability.

“I think I have not returned to being the same as before, let us put it that way, but I do not suffer, you know, I do not have still-lingering pains”.

The experience of a fetal demise also marks a change in self-perception for Stefania, 36 years old. The collapse of the certainty that life can be controlled in every aspect leads to a newfound awareness.

“Well, there has been a before and an after. I have always been a very cheerful and light-hearted person […] I became more, more serious, darker, and I don’t know as if that… I mean, that innocence is missing, I do not know how to explain it, but yes, there has been a moment of a tough change in my character, in life.”

For some mothers, new possibilities and paths in life emerge, along with personal growth and self-discovery. This is evident in Anna’s narration, as she states:

“I would describe myself as going through a process of rebirth. After what happened to me twice, especially this time, I believe I am on a journey of rebirth, reevaluating my body, my mind, and even my perspective on life, as well as my relationship with my husband. I think that, in this journey, I could improve myself as a woman, as a person, in my relationships with others and with life.”

These narratives demonstrate how the loss experienced by these individuals has brought about profound changes in their self-perception, leading to a process of personal growth and transformation.

#### 3.2.2. Conflicted Relationship with One’s Body

Due to its “embodied” nature, pregnancy loss can give rise to new representations of the body. The body, which was once seen as a vessel of life, now becomes associated with generating death. Within the group of women in the study, some experience a sense of failure in their bodies, feeling betrayed and abandoned. With the word of Giulia:

“The first time, I felt betrayed by my body. Um, I felt deceived, I do not know why. I have always been careful about health, nutrition. I have always been very attentive to my body, taking great care to go for check-ups and to take care of myself in the best possible way, so I felt betrayed, abandoned […]. This time, I felt it even more. After the second miscarriage, I said to myself, ‘but why again after all the attention I’ve paid, even more in this pregnancy […].’ This time I felt betrayed by my body once again, as if I were fundamentally wrong.”

Feelings of betrayal can last in time and influence future experiences of attempting to get pregnant. Two years after the loss due to a spontaneous abortion, Gaia says she hates her body because despite the time that has passed, it has not allowed her to become a mother.

“Then when we decided to continue our search and it was not happening, I had a bit, how to say it, of hatred for my body, ‘It does not work!’ […]. The fact that after two years I have not been able to get pregnant again, and so the relationship with your body after these two years is difficult.”

In certain narratives, a prevailing sentiment is one of guilt due to their perceived failure as caregivers, unable to carry the pregnancy to term. This negatively impacts their sense of self-efficacy and self-esteem. From these accounts, a view of a flawed body that does not function properly emerges.

“Even now, there are days when I do not accept and like myself, especially at the beginning, there was a bit of resentment towards my body, as if to say ‘you could not’ carry the pregnancy to term, or there are still things not working, things to fix’; so, even there, a very fluctuating relationship. There are days when I accept it as it is […] there are days when instead it’s as if I have a bit of this resentment towards my body, right? For not succeeding in the attempt.”

Therefore, in some narratives, the pain of loss becomes inherently intertwined with a perceived failure to meet societal expectations related to the role of caregiving and parental protection. This can have a detrimental impact on the mother’s identity and self-perception. It is important to acknowledge and address these complex feelings and challenges individuals face in reconciling their relationship with their bodies after experiencing pregnancy loss.

#### 3.2.3. Negative Impact of COVID-19 vs. Unexpectedly Positive Aspects

The isolation resulting from COVID-19 restrictions has had a profound impact on the lives of two mothers, Stefania and Chiara. Both express the need to regain a sense of normalcy, which includes going out and socializing with friends. For Stefania, the easing of the pandemic situation in 2021 was of great help in processing her loss. She states:

“Currently, one year later, I would say that normalcy has almost returned. With work commitments, social engagements, and the general reopening, it has allowed us to shift our focus away from that moment […] we have returned to somewhat of our regular lives.”

During follow-up interviews, some women express concerns about COVID-19 vaccination. Stefania and Anna’s narratives strongly highlight the fear of not being able to conceive after their loss. For Stefania, vaccination was a compulsory choice due to her job. She describes herself as perplexed and doubtful about the safety of the vaccine and its effects.

However, some stories emphasize the surprising benefits of the pandemic situation, such as increased opportunities for bonding with children and partners and restrictions on home and hospital visits. In Susana’s account, the COVID-19-induced isolation was somewhat enjoyable, as she states:

“It was just the three of us at home: me, my husband, and my stepdaughter, who moved in because she also had COVID-19. We painted the house, rearranged all the rooms, and did some home improvements, so it was also fun.”

These narratives showcase both the negative impact of COVID-19 restrictions, particularly in terms of isolation and concerns about vaccination, as well as the unexpectedly positive aspects, such as quality time with family and engaging in home projects.

## 4. Discussion

The present study offers an update of data available at a baseline and data from a six-month follow-up regarding the psychological impacts of perinatal death, specifically occurring during times of health-related public restrictions due to COVID-19. Consequently, the study provides healthcare services, practitioners, and the wider community, with relevant insights into coping with traumatic losses amidst social isolation and. Moreover, clinicians and healthcare personnels could benefit from detailed parental accounts to gain a more person-centered perspective on the significance of such losses.

The analysis of the self-report questionnaire (PG-13), administered six months later, confirmed the absence of a diagnosis of complicated grief in the participants [33]. Furthermore, during the baseline phase of the study, quantitative data confirmed the presence of symptomatology related to post-traumatic stress disorder in 50% of the participants, while 20% suffered from relational dyadic stress, thus showing low levels of couple satisfaction and disturbances in the parental affective sphere [24]. In the follow-up, quantitative results showed that 84% of the participants displayed symptomatology related to post-traumatic stress disorder, indicating an increased incidence of the disorder in the population after six months. This significant finding could underscore the traumatic potential of losing a child. The existing literature suggests that one in four women is at risk of PTSD following a loss event [49,50]. Furthermore, perinatal loss, particularly stillbirth, profoundly affects parental couples [51] with identity-related consequences. Their parental project is abruptly interrupted, along with their identity as mother and father, as evidenced by emerging themes in biographical narratives after six months [15].

The trauma of loss often leads women to develop a challenging relationship with their bodies. The third theme confirms the profound impact of perinatal loss on women (miscarriage, spontaneous abortion) due to its “embodied” nature [1,2,3]. Many women report feeling their bodies have failed them after experiencing loss, perceiving a disruption in their femininity, as confirmed by studies on grieving mothers [13]. In some cases, their narratives reveal a sense of guilt caused by their perceived failure as caregivers [14]. For many women, motherhood holds significant meaning and expectations, and the perceived flawed in their bodies impacts their self-perception and overall self-evaluation [52,53].

There is a notable shift in maternal and paternal affectivity, significantly diminished after six months, resulting in decreased anxiety, depression, perceived stress, anger, and relational problems. This finding appears to contradict the increased symptomatology attributed to PTSD. However, the perceived support from the parental couple can deeply influence the post-loss experience and the ability to integrate the loss event into the biographical narrative, as indicated by the accounts of interviewed mothers and fathers. They are often the protagonists of delegitimized grief, which causes profound suffering due to the invalidation of their pain and the lack of acknowledgment of the loss they have experienced [12]. The narratives highlight the importance for the parental couple to share their experience with mothers and fathers who have had similar experiences. As confirmed by the literature, participation in support groups has a positive effect on traumatic stress response as it promotes sharing. Connecting with other women, often experiencing delegitimized grief, compensates for the lack of recognition of their loss by the pre-loss social group [54].

Correlational analysis revealed a significant relationship between the new instrument in the follow-up, the ICSG, and the two tools presented in the baseline phase, namely the PG-13 and the PAPA/PAMA. The higher the level of spiritual crisis in the participants (problematic relationship with God and community members), the more they showed anxiety and difficulty processing the loss event. In particular, the strongest positive correlation between the “Disruption of Religious Practice” subscale and the PAMA/PAPA reveals a greater distance from the community of believers compared to the reserved relationship with God (see “Insecurity with God” subscale). Similarly, the more participants experienced a religious crisis post-loss, the higher the presence of anxiety, depression, perceived stress, anger, and relational problems. This result confirms the relationship between negative religious coping and symptoms related to depression, anxiety, and relational issues. High levels of spiritual distress, particularly regarding the relation with a lost and shattered God, are associated with higher anxiety levels and post-loss stress [55]. Furthermore, this finding is reinforced by the narratives of several mothers who report distancing themselves from religious practice and experiencing a profound rupture in their relationship with God following the traumatic event.

One year after the loss event, participants actively seek meaning to attribute to their pain. Some reframe the loss event, attempting to integrate it into their biographical narratives, as confirmed by the literature on the grief of parents who have survived traumatic deaths [56]. Many participants resort to scientific and religious interpretations as veiled attempts to find a reason for the loss [57]. However, due to the limited participant pool, conducting a more comprehensive statistical analysis was not feasible, and the findings cannot be broadly applied. Additionally, the majority of the participants are Italian, hold university degrees, and belong to medium-to-high economic brackets, rendering the results non-representative of a broader demographic.

Contrary to the quantitative result indicating increased PTSD among participants, their narratives present domains related to post-traumatic growth. Following the collapse of their assumptive world, the responses of many participants reveal a change in life priorities. Some show a greater appreciation for life, while others value the existential dimension. Others express a desire to nurture closer relationships with others. Still, others feel the need to prioritize and value their relationship with their partner by dedicating more time to it. After six months from the baseline phase, many parents exhibit changes that go beyond their initial condition, manifesting a generative change in their lives [58].

Finally, results of the follow-up confirm the impact of the pandemic situation on the experience of the parental couple. On the one hand, participants expressed many indirect effects created by the pandemic condition and public health restrictive measures, such as difficulties in accessing healthcare facilities, lack of adequate support from healthcare personnel, and fathers’ inability to assist and offer support during their wives’ hospitalization. On the other hand, some participants expressed doubts, perplexities, and fears about the effects of vaccination. In particular, there is fear of failing to conceive again or causing long-term illnesses in the child, especially in cases such as subsequent pregnancies after the loss. Additionally, mothers and fathers, due to contagion restrictions, were forced to isolate themselves without being able to go out and distract themselves. This condition negatively affected the process of processing the loss. Mothers had to attend medical visits alone and often interacted with unsympathetic healthcare personnel, causing them profound distress [59]. However, qualitative analysis reveals surprising benefits related to the pandemic situation, especially in the perinatal and post-loss experiences [30]. The low rate of fathers’ participation and involvement in the research is problematic and limits the generalizability of the findings on that population. Many cultural factors are associated with the expression of grief. Moreover, during the hospitalization period, many fathers continued to work as well as manage daily home duties and thus were still exposed to many activities that could have impacted their willingness to participate.

Training for healthcare personnel and the inclusion of psychologists in gynecological and obstetric teams are crucial, especially in complex situations like a pandemic. Promoting cognitive-behavioral strategies and activities like journaling and mindfulness is essential, benefiting women’s psychological health. Limitations of this study include a lower number of fathers, a small participant group due to non-adherence, and limited generalizability due to geographic and sociodemographic constraints. Moreover, biases could have intervened in self-report measures as well as during the interviews. However, the disenfranchised nature of such a loss, as discussed in the Introduction section, makes it possible to consider that people who participated were genuinely willing to share their experiences. Further studies could explore the relationship between religiosity/spirituality and post-traumatic growth, as thematic analysis revealed potential connections with participants’ perceptions of transcendence [60]. Moreover, longitudinal studies could explore in more detail the evolution of grief and coping over time as well as relevant factors promoting the grief work on such a loss. In particular, clinical psychological or psychosocial interventions, such as mutual self-help groups, inspired by meaning-making perspectives could help individuals to intersubjectively and collectively reframe the grieving experience and share useful psychological resources to cope with such a loss [61,62]. Indeed, qualitative results confirm that the adaptation of one’s own life to perinatal loss requires a constant reframing of personal meanings and values, as reflected in the parental project, the body, and the relationship with the spiritual dimensions. Moreover, specific interventions directed to the management of the most intrusive and dysfunctional symptoms could apport brief-term results, and a more structured clinical intervention could be developed.

## 5. Conclusions

The baseline findings indicated that COVID-19 restrictions have adversely impacted the experience of spontaneous pregnancy loss in couples [5], with follow-up results confirming the lasting detrimental effects on the processing of traumatic loss. These restrictions have strained relationships between healthcare providers and parents, limiting interactions with the family and friends network. Inadequate psychological services exacerbated psychological distress in couples, evidenced by 84% exhibiting symptoms of post-traumatic stress disorder six months post-loss. Follow-up interviews revealed participants’ reports of dehumanizing treatments during hospitalization and post-loss check-ups, emphasizing the significant impact of healthcare staff communication on long-term well-being.

## Figures and Tables

**Table 1:** Participants’ characteristics (n = 45).

Variable ^1^	Baseline (n = 45)	Follow-Up (n = 20)
**Gender**		
Female	37 (82%)	13 (65%)
Male	8 (18%)	7 (35%)
**Age**	28–47; 36.56 (4.14)	29–48; 37.60 (4.69)
**Nationality**		
Italian	39 (87%)	17 (85%)
Other	6 (13%)	3 (15%)
**Education**		
High school or lower	22 (49%)	5 (25%)
Bachelor’s or master’s degree	23 (51%)	15 (75%)
**Occupation**		
Worker	38 (84%)	20 (100%)
Unemployed	5 (11%)	0 (0%)
Housewife	2 (4%)	0 (0%)
**Marital Status**		
Married/cohabiting	44 (98%)	20 (100%)
Separated/divorced	1 (2%)	0 (0%)
**Economic Level**		
Low level	27 (60%)	10 (50%)
Medium or high level	18 (40%)	10 (50%)
**Previous Children**		
No	20 (44%)	14 (70%)
Yes	20 (56%)	6 (30%)
**Pregnancy Variables**		
Gestational week	2–42; 17.64 (10.69)	2–42; 19.20 (11.87)
Number of previous pregnancies	0–5; 1.22 (1.28)	0–5; 0.85 (1.23)
**Previous Miscarriages/Stillbirth**		
No	28 (62%)	12 (60%)
Yes	17 (38%)	8 (40%)
**Percived Support**		
Support from family	1–5; 3.53 (1.31)	1–5; 3.55 (1.19)
Support from friends	1–5; 3.73 (1.10)	1–5; 3.65 (0.81)
Support from health services	1–5; 3.60 (1.30)	1–5; 3.35 (1.04)
**Support from Psychologist**		
No	31 (69%)	12 (60%)
Yes	14 (31%)	8 (40%)

^1^ Data are expressed as range; means and standard deviation (in brackets) for continuous variables; and as frequency and percentage (in brackets) for categorical variables.

**Table 2:** Cronbach’s alpha of each adopted instrument at the baseline and at the follow-up.

Measure	Baseline (n = 45)	Follow-Up (n = 20)
IES-R Total	0.89	0.88
IES-R Avoidance	0.75	0.75
IES-R Intrusiveness	0.85	0.80
IES-R Hyperarousal	0.72	0.63
PG13 Total	0.84	0.75
PAMA/PAPA Total	0.67	0.79
DAS-4 Total	0.60	0.59
DSES Total	0.93	0.95
ICGS Total	Not present	0.87

**Table 3:** Descriptive statistics and gender differences at baseline (n = 45).

Measure	Mothers (n = 37)	Fathers (n = 8)	*p*	Effect Size of Differences ^1^
Distress measures:				
IES-R Total	31.95 (16.93)	25.63 (13.44)	0.365	0.21
IES-R Avoidance	1.35 (0.81)	1.17 (0.82)	0.494	0.16
IES-R Intrusiveness	1.62 (1.01)	1.28 (0.92)	0.457	0.17
IES-R Hyperarousal	1.36 (0.89)	1.00 (0.74)	0.205	0.29
PG13 Total	25.68 (8.57)	22.75 (7.32)	0.319	0.23
IES-R PTSD (1 = Yes; 0 = No)	18 (49%)	3 (37%)	0.567	0.08
PG13 DLP (Yes = 1; 0 = No)	1 (3%)	0 (0%)	0.638	0.07
Perinatal Maternal/Paternal Affectivity:				
PAMA/PAPA Total	36.35 (14.21)	29.63 (19.68)	0.373	0.21
Couple Satisfaction:				
DAS-4 Total	14.14 (4.12)	13.88 (2.17)	0.346	0.22
DAS-4 Distressed spouse (Yes = 1; No = 0)	13 (35%)	3 (37%)	0.899	−0.02
Spirituality:				
DSES Total	56.16 (19.71)	48.88 (13.71)	0.350	0.22

^1^ Effect sizes used are biserial rank correlation for continuous variables and tetrachoric correlation for dichotomous variables.

**Table 4:** Multiple regression analysis for distress measures at baseline (n = 45).

Predictor ^1^	IES-R				PG-13
	Total	Avoidance	Intrusiveness	Hyperarousal	Total
Participants characteristics:					
Gender (Female = 1; Male = 0)	0.24	0.10	0.28	0.20	0.00
Age	−0.05	−0.05	−0.11	0.08	−0.13
Nationality (Italian = 1; Other = 0)	0.14	0.08	0.11	0.16	−0.11
Education (High = 1; Lower level = 0)	−0.15	−0.11	−0.09	−0.17	0.01
Occupation (Worker = 1; Other = 0)	0.19	0.00	0.35 ~	0.06	0.05
Economic Condition (Medium-High = 1; Lower level = 0)	0.21	0.05	0.37 *	0.03	0.04
Gestational week	0.39 *	0.23	0.36 *	0.38 *	0.30 *
Number of previous pregnancies	0.11	0.23	−0.02	0.08	0.10
Previous miscarriages (Yes = 1; No = 0)	−0.05	−0.36	0.05	0.22	−0.13
Support from family	−0.07	−0.09	−0.14	0.11	0.02
Support from friends	−0.08	0.00	−0.06	−0.15	−0.10
Support from health services	−0.32 ~	−0.38 ~	−0.25	−0.15	0.01
Perinatal Maternal/Paternal Affectivity:					
PAMA/PAPA Total	0.50 *	0.50 *	0.35 ~	0.41 ~	0.67 **
Couple Satisfaction:					
DAS-4 Total	−0.09	0.13	−0.32 ~	0.05	−0.11
Spirituality:					
DSES Total	−0.05	0.14	−0.19	−0.02	−0.01
R-square	48%	41%	57%	40%	65%

^1^ Data are expressed as standardized regression coefficients (beta). ~ *p* < 0.10; * *p* < 0.05; ** *p* < 0.01.

**Table 5:** Baseline and 6-month follow-up comparison (n = 20).

Measure ^1^	Baseline	Follow-Up	t (19)	*p*	Effect Size of Differences (Cohen’s d)
Distress measures:					
IES-R Total	29.55 (12.92)	19.40 (12.83)	4.67 ***	<0.001	1.04
IES-R Avoidance	1.25 (0.82)	0.91 (0.69)	3.53 **	0.002	0.79
IES-R Intrusiveness	1.56 (0.80)	1.04 (0.74)	3.28 **	0.004	0.73
IES-R Hyperarousal	1.17 (0.63)	0.62 (0.52)	4.00 **	0.001	0.90
PG13 Total	24.65 (8.70)	19.15 (4.88)	3.61 **	0.002	0.81
Perinatal Maternal/Paternal affectivity:					
PAMA/PAPA Total	35.15 (14.06)	19.90 (15.71)	5.31 ***	<0.001	1.19
Couple Satisfaction:					
DAS-4 Total	14.70 (2.39)	15.10 (2.15)	−0.65	0.525	−0.14
Spirituality:					
DSES Total	55.05 (17.07)	54.55 (19.08)	0,27	0.792	0.06

^1^ Data are expressed as means and standard deviation (in brackets) ** *p* < 0.01; *** *p* < 0.001.

## Data Availability

Data are contained within the article.
